# Effective long-term sirolimus treatment in hypoxemia mainly due to intrapulmonary right-to-left shunt in a patient with multiple vascular anomalies

**DOI:** 10.1186/s13023-023-02732-3

**Published:** 2023-05-24

**Authors:** Jinrong Liu, Xiaomin Duan, Jie Yin, Haiming Yang, Ruxuan He, Shunying Zhao

**Affiliations:** 1 Department of Respiratory Medicine, National Clinical Research Center of Respiratory Disease, Beijing Children’s Hospital, National Center for Children’s Health, Capital Medical University, Beijing, China; 2 Imaging Center, Beijing Children’s Hospital, National Center for Children’s Health, Capital Medical University, Beijing, China

**Keywords:** Sirolimus, Hypoxemia, Intrapulmonary, Right-to-left shunt, Arteriovenous malformation, Patent ductus venosus, Children

## Abstract

Pulmonary arteriovenous malformations (PAVMs), particularly where feeding artery/arteries to PAVMs ≥ 3 mm can be treated with embolization. The treatment for hypoxemia resulting from multiple small or diffuse PAVMs remains unclear.

We report a girl aged 5 years and 10 months presented with cyanosis and decreased activity after exercise (83–85% of pulse oxygen saturation, SpO_2_). She had 1 skin lesion on her face and 1 suspected hemangioma on her left upper extremity at birth and that gradually disappeared spontaneously. Physical examination revealed clubbed fingers, and abundant vascular networks on her back. Contrast-enhanced lung CT (slice thickness:1.25 mm) with vascular three-dimensional reconstruction and abdominal CT revealed increased bronchovascular bundles, increased diameter of the pulmonary artery and ascending aorta, and intrahepatic portosystemic venous shunts due to patent ductus venosus. Echocardiography revealed increased diameter of aortic and pulmonary artery. Transthoracic contrast echocardiography was highly positive (bubble appearing in the left ventricle after 5 cardiac cycles). Abdominal doppler ultrasound revealed hepatic-portal venous shunt. Magnetic resonance imaging, artery and vein of the brain revealed multiple malformations of venous sinuses. The patient received sirolimus for 2 years and 4 months. Her condition improved significantly. SpO_2_ gradually increased to 98%. Her finger clubbing gradually normalized.

Our report implicates sirolimus might be a potential treatment option in persistent hypoxemia mainly due to intrapulmonary right-to-left shunt even small multiple or diffusive PAVMs in pediatric patients with multiple cutaneous and visceral vascular anomalies.

## Introduction

Anatomical intrapulmonary right-to-left shunt is mainly caused by pulmonary arteriovenous malformations (AVMs) in Children. Pulmonary AVMs (PAVMs) are structurally abnormal, direct vascular communications between pulmonary arteries and veins, which bypass the normal pulmonary capillary beds and result in an intrapulmonary right-to-left shunt. PAVMs can be single or multiple, simple or complex, and unilateral or bilateral [1]. PAVMs, particularly multiple and/or diffuse PAVMs, can be part of a syndrome, often familial, such as hereditary hemorrhagic telangiectasia (HHT) [1, 2]. Acquired PAVMs have been reported in hepatopulmonary syndrome (HPS) and patent ductus venosus (PDV) [3–5]. PAVMs are most frequently asymptomatic, but may be associated with hemoptysis, dyspnea, and hypoxemia due to paradoxical embolization. If left untreated, PAVMs can result in life-threatening complications such as massive hemoptysis, stroke and brain abscesses [6]. PAVMs particularly focal PAVMs with feeding arteries at least 3 mm in diameter can be treated with embolization [6–8]. 25% of children with HHT whose PAVMs were considered too small to require embolization at initial screening went on to develop lesions large enough for intervention within 3–5 years [9]. Three primary targeted anti-angiogenic therapies including intravenous bevacizumab, oral pazopanib and oral thalidomide have been reported to treat the symptoms mainly nasal and gastrointestinal bleeding in adults with HHT [10, 11]. Both pulmonary artery (PA) hypertension (PAH) and HHT have been treated with sirolimus in case reports. However, the treatment of hypoxemia resulting from multiple small PAVMs remains unclear.

In this study, we report on a pediatric female patient with highly positive bubble echo with a negative CT-enhancement (slice thickness: 1.25 mm), suggesting microPAVMs < 1.25 mm in size, who predominantly presented with persistent hypoxemia and was effectively treated with long-term sirolimus/rapamycin.

### Case presentation

**Fig. 1:**
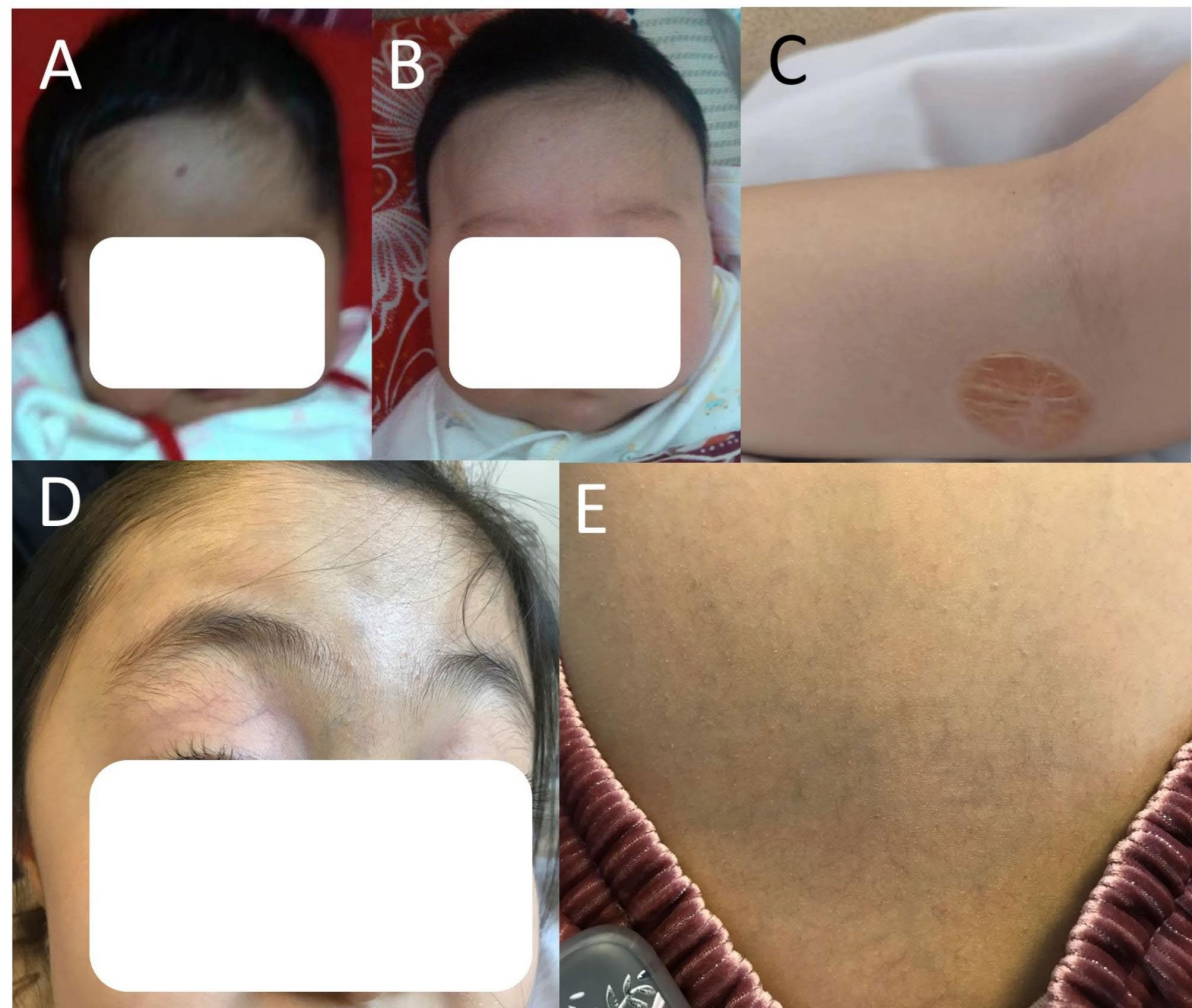
Dark red skin/vascular lesion (3 × 2 mm) on her face (**A**) at birth and 2 months of age (**B**). Fibrofatty residua from an involuted infantile hemangioma on the left upper extremity (**C**). Abundant vascular networks on the upper eyelid (**D**) and back (**E**)

A girl, aged 5 years and 10 months, presented with a 2-year history of cyanosis and decreased activity after exercise (83–85% of pulse oxygen saturation, SpO_2_). Three months before admission, she had developed respiratory failure associated with bronchitis, and SpO_2_ decreased to 77–80%. Past history revealed that she had had 1 dark red skin/vascular lesion (3 × 2 mm) on her face (Fig. 1A) and 1 bright red puffy suspected hemangioma (30 × 30 mm) on her left upper extremity at birth and that gradually had disappeared spontaneously (Fig. 1B). Between them, fibrofatty residua had been left on her left upper extremity (Fig. 1C). One suspected hemangioma within the placenta (65 mm×55 mm) was found by doppler during pregnancy and was visible during delivery in her mother. Physical exam revealed 1 pinpoint-sized telangiectasia on the left upper extremity of her father (age 35) and no telangiectasias on her mother (age 33). Her family history revealed her maternal grandparents and paternal grandmother had had hypertension. The family members had never experienced nosebleeds. SpO_2_ was 100% in both of her parents.

**Fig. 2:**
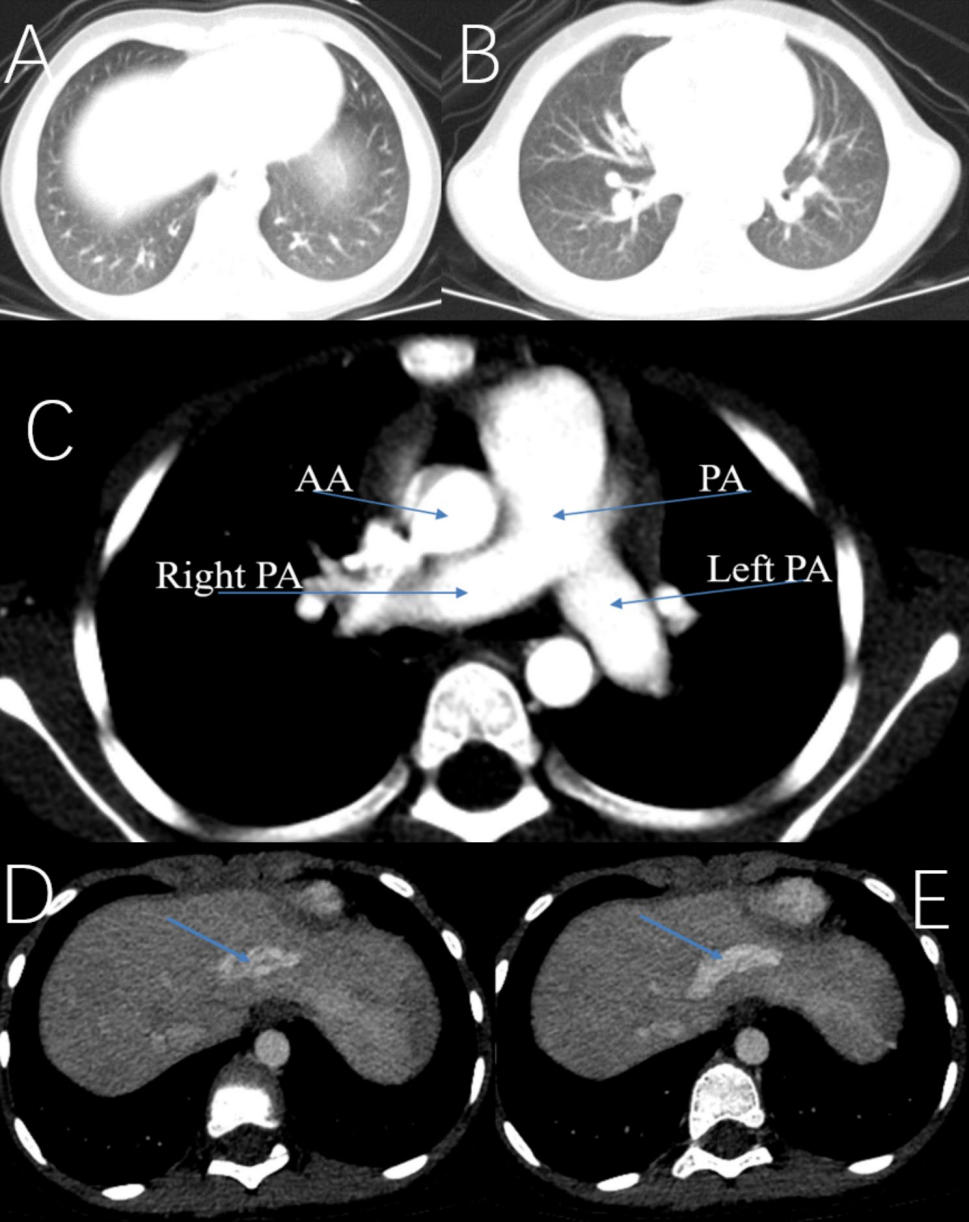
Contrast enhanced lung CT with vascular three-dimensional reconstruction revealed increased pulmonary vascular bundles and bronchovascular bundles (**A, B**), increased diameter of the pulmonary artery (PA), left PA right PA and ascending aorta (AA) (**C**), and left hepatic venous-left portal venous shunt (**D, E**)

**Fig. 3:**
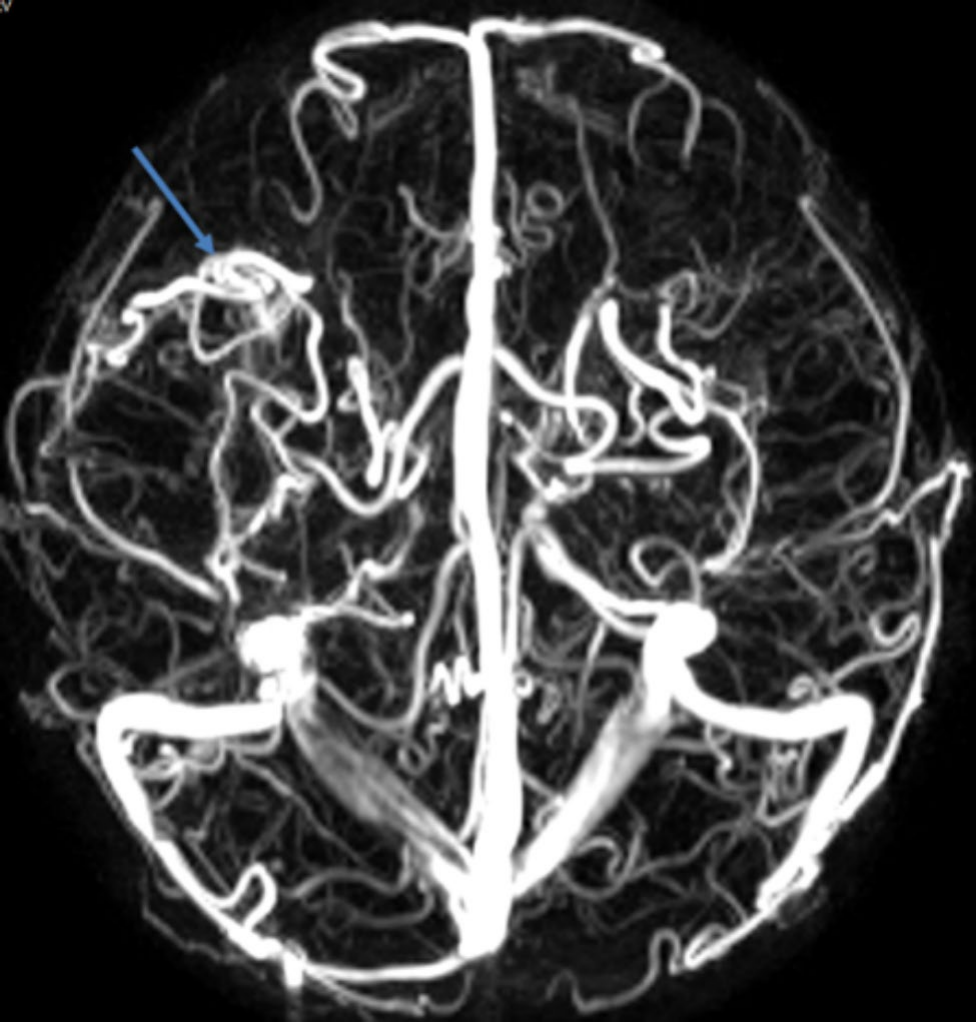
Non-contrast magnetic resonance imaging, artery and vein of the brain revealed multiple malformations of venous sinuses

Physical examination revealed cyanosis of nail beds, clubbed fingers, and abundant vascular networks on her face particularly upper eyelid and on her back (Fig. 1D and E). Her body mass index was 13.1. Arterial blood gas analysis revealed a pressure of arterial oxygen of 55 mmHg and an oxygen saturation of 87% of in the supine position. In laboratory investigations, we detected normal liver enzymes, renal function and ceruloplasmin; and increased serum lactate dehydrogenase (LDH), indirect bilirubin (IBIL), total bile acids (TBA), and glycocholic acid (GA) (Table 1). Coombs’ test was negative. Screening for metabolic diseases revealed no abnormalities. Contrast enhanced lung CT with vascular three-dimensional reconstruction and abdominal CT revealed increased pulmonary vascular bundles and bronchovascular bundles (Fig. 2A and B); increased diameter of the main PA (22 mm), left PA (16 mm), right PA (15 mm) and ascending aorta (AA, 14 mm) (Fig. 2C); and left hepatic venous-left portal venous shunt (Fig. 2D and E; Table 2). Doppler echocardiography revealed increased diameter of aortic and pulmonary artery, and mild tricuspid and pulmonary valve regurgitation with a tricuspid regurgitation pressure gradient (TRPG) of 19.7 mmHg. Transthoracic contrast echocardiography (TTCE) was positive with grade 3/3 (bubble appearing in the left ventricle after 5 cardiac cycles). Pulmonary perfusion scan showed radioactivity in the brain, bilateral kidneys, liver, and spleen, suggesting the existence of intrapulmonary shunt due to PAVMs (shunt: 12.8%, Table 2). Abdominal and portal vein system color doppler ultrasound revealed a tortuous and dilated tubular structure connecting the left hepatic vein with the left branch of portal vein in liver suggesting left hepatic venous-left anterior branch of portal venous shunt (Table 2), and normal liver elasticity (4.64kpa). Interventional arteriography was performed, but no intrahepatic arteriovenous shunt was found. Non-contrast magnetic resonance imaging, artery and vein of the brain revealed multiple malformations of venous sinuses (Fig. 3). Using trio whole-exome sequencing (WES), we did not identify a variant [including an intronic variant that is > 15 bp from the exon boundaries (which are unlikely to affect messenger RNA splicing)], or a large genomic deletion associated with vascular malformations (including *ENG, ACVRL1, SMAD4, GDF2, RASA1*, and *EPHB4* genes). The other results of auxiliary examination are summarized in Tables 1 and 2.

**Table 1:** Laboratory findings of the patient with multiple vascular anomalies

Markers in peripheral blood	Actual value	Normal Range
RBC (×10^12^/L)	4.77–5.85	4.3–5.7
Hemoglobin(g/dL)	14.8–17.1	12.1–15.8
Platelet (×10^9^/L)	167–185	177–446
LDH (IU/L)	281–434	110–295
IBIL (µmol/L)	25–40	0–17
TBA (µmol/L)	38–143	0–10
GA (mg/L)	39–68	0-2.7

**Abbreviations**: RBC-red blood cell; LDH-lactate dehydrogenase; IBIL-indirect bilirubin concentration; TBA-total bile acid; GA- glycocholic acid

**Table 2:** The dynamic findings of abdominal doppler and pulmonary perfusion scan

Abdominal doppler		On admission		1 year on treatment		2 years on treatment
left hepatic venous-left portal venous shunt (mm)		15 × 8 × 26		11 × 5 × 19		12 × 6 × 18
**Pulmonary perfusion scan**		**On admission**		**6 months on treatment**		**1 year on treatment**
Shunted organs		brain, bilateral kidneys, liver, and spleen		-		-
shunt rate (%):		12.8		4.85		4.09
Pulmonary nodule		Left lower		-		-

The patient received treatment with intermittent low-flow supplemental oxygen and sirolimus (0.8-1 mg/m^2^ once daily orally) for a goal trough level of 8–15 ng/Ml [12]. Her physical strength and cyanosis improved significantly after 2 weeks on treatment. Her finger clubbing improved significantly after 1 month on treatment and gradually normalized. Hepatic venous-portal venous doppler shunt improved (Table 2), and sirolimus was held for 20 days due to elevated alanine aminotransferase and increasing IBIL after 1 year on treatment. At the age of 8 years, sirolimus was discontinued after 2 years and 4 months of treatment when our patient received Covid-19 vaccine.

Currently, at the age of 9 years, serum vascular endothelial growth factor (VEGF) was detected and revealed a very high level of 1485pg/ml (R&D, DVE00). The serum VEGF concentrations were 56pg/ml and 32pg/ml in her mother and father, respectively. From the age of 7 up to now, SpO_2_ was 98% or so, and she could walk 10,000 steps every day, and ran 30–50 m every time (her parents prevented her from running longer distances). Therefore, we did not prescribe sirolimus for her again.

## Discussion

Vascular anomalies are a diverse group of disorders resulting in morbid and life-threatening complications. PAVMs are rare and most frequently congenital. It is estimated that at least 80% of PAVMs are associated with HHT [13]. PAVMs may be the only clinical criterion present in genetically confirmed HHT [14]. Combined PAVMs and portal hepatic venous shunt in HHT [15], and PAVMs in *RASA1*-related capillary malformation-AVM (CM-AVM1) [16] have been reported, so HHT and CM-AVMs were suspected on her, however WES did not detect a variant. HPS was also suspected, because of intrahepatic portosystemic venous shunts due to rare congenital PDV [17], and persistent elevations of serum IBIL, TBA and GA in our patient. SpO_2_ was as low as 83–85%, pulmonary perfusion scan showed a significant right-left shunt of 12.8%, and TTCE grading was 3/3, suggesting a single large or many small PAVMs [13]. In the absence of a visible large PAVM on CT, we speculated that there were multiple small or even diffuse PAVMs in our patient. There is no ideal cure for diffuse small PAVMs, and persistent hypoxemia severely affects pediatric patient’s development, mental health, and quality of life. Additionally, respiratory failure was severe in the setting of lower respiratory tract infection.

Excessive angiogenesis is a key pathogenesis in HHT, CM-AVM, HPS, and some other vascular malformations [18–21]. PI3K signaling is increased and stimulates vascular endothelial cell proliferation in mouse models of HHT associated AVMs and cerebral cavernous malformations (CCMs) [19, 22] by various angiogenic growth factors including VEGF, a key regulator of angiogenesis and lymphangiogenesis [22]. PI3K inhibition efficiently prevents AVM formation and reverts established AVMs and improves aggressive CCMs [19, 22]. Sirolimus is a specific and potent inhibitor of mTOR, a serine/threonine kinase in the PI3K/Akt pathway. Sirolimus can target vascular cell proliferation and it is effective for vascular anomalies such as complicated and refractory infantile hemangiomas, combined hemangiomas and pulmonary hypertension, and pulmonary vein stenosis [12, 23–25]. In addition, high-dose sirolimus has been shown to improve portal hypertension and decrease intrapulmonary shunting through inhibition of the mTOR/VEGF, and NFκB signaling pathway in cirrhotic-HPS rats [26].

Our patient had multiple cutaneous and visceral vascular anomalies, which suggested she might be syndromic. Additionally, a very high level of serum VEGF suggested an excessive angiogenesis on her. Our previous study reported that sirolimus improved hypoxemia due to diffuse PAVMs in 1 boy with *GDF2*-related HHT after 14 days on treatment [2]. Subsequently, however, his SpO_2_ started to decrease, and sirolimus was discontinued after 3 months of treatment. Ruiz et al. reported that the combination of sirolimus and nintedanib concurrently could normalize endothelial Smad1/5/8, mTOR, and VEGFR2 pathways to synergistically and efficiently oppose the associated vascular pathology in HHT mouse models [27], which may explain the treatment failure of sirolimus alone in our previous study [2]. Doppler echocardiography revealed a normal TRPG, which suggested that PA pressure was normal in our present patient. However, PDV may cause PAH [28], the PA/AA ratio was increased, and PAVMs may offset the PA hypertension, which suggested a possible potential PAH on her. Sirolimus may prevent PAH [23, 29]. Our present patient ultimately started treatment with sirolimus. Fortunately, her symptoms significantly improved and she could have normal activity level and quality of life for her age. She received more than 2 years of treatment with sirolimus, which further supports the efficacy and safety of sirolimus in vascular anomalies.

Our study has several limitations. We did not undertake pulmonary angiography. Blood gas analysis and TTCE were not monitored after treatment. Many questions remain around the use of sirolimus in our patient and in vascular anomalies in general, including duration of therapy and appropriate trough level targets.

## Conclusions

To the best of our knowledge, this is an important report of effective long-term sirolimus treatment in persistent hypoxemia mainly due to intrapulmonary right-to-left shunt even small multiple PAVMs in a pediatric patient with multiple cutaneous and visceral vascular anomalies and a very high level of serum VEGF.

## Data Availability

The datasets analyzed for this study are available from the first author Dr. Jinrong Liu (liujinrong2006@163.com) upon reasonable request.

## Abbreviations

AA: Ascending aorta
AVMs: Arteriovenous malformations
CCMs: Cerebral cavernous malformations
CM: AVM–capillary malformation-arteriovenous malformation
GA: Glycocholic acid
HHT: Hereditary hemorrhagic telangiectasia
HPS: Hepatopulmonary syndrome
IBL: Indirect bilirubin
LDH: Lactate dehydrogenase
PA: Pulmonary artery
PAH: Pulmonary artery hypertension
PAVMs: Pulmonary arteriovenous malformations
PDV: Patent ductus venosus
SpO_2_: Pulse oxygen saturation
TBA: Total bile acids
TRPG: Tricuspid regurgitation pressure gradient
TTCE: Transthoracic contrast echocardiography
VEGF: Vascular endothelial growth factor
WES: Whole exome sequencing
